# Changes in Antibody Levels during and following an Episode of Acute Adenolymphangitis (ADL) among Lymphedema Patients in Léogâne, Haiti

**DOI:** 10.1371/journal.pone.0141047

**Published:** 2015-10-22

**Authors:** Katherine E. Mues, Patrick J. Lammie, Mitchel Klein, David G. Kleinbaum, David Addiss, LeAnne M. Fox

**Affiliations:** 1 Department of Epidemiology, Rollins School of Public Health and Laney Graduate School, Emory University, Atlanta, Georgia, United States of America; 2 Parasitic Diseases Branch, Division of Parasitic Diseases and Malaria, Center for Global Health, Centers for Disease Control and Prevention, Atlanta, Georgia, United States of America; 3 Task Force for Global Health, Decatur, Georgia, United States of America; Brighton and Sussex Medical School, UNITED KINGDOM

## Abstract

**Introduction:**

Episodes of acute adenolymphangitis (ADL) are often the first clinical sign of lymphatic filariasis (LF). They are often accompanied by swelling of the affected limb, inflammation, fever, and general malaise and lead to the progression of lymphedema. Although ADL episodes have been studied for a century or more, questions still remain as to their etiology. We quantified antibody levels to pathogens that potentially contribute to ADL episodes during and after an episode among lymphedema patients in Léogâne, Haiti. We estimated the proportion of ADL episodes hypothesized to be attributed to specific pathogens.

**Methods:**

We measured antibody levels to specific pathogens during and following an ADL episode among 41 lymphedema patients enrolled in a cohort study in Léogâne, Haiti. We calculated the absolute and relative changes in antibody levels between the ADL and convalescent time points. We calculated the proportion of episodes that demonstrated a two-fold increase in antibody level for several bacterial, fungal, and filarial pathogens.

**Results:**

Our results showed the greatest proportion of two-fold changes in antibody levels for the carbohydrate antigen *Streptococcus* group A, followed by IgG2 responses to a soluble filarial antigen (BpG2), Streptococcal Pyrogenic Exotoxin B, and an antigen for the fungal pathogen *Candida*. When comparing the median antibody level during the ADL episode to the median antibody level at the convalescent time point, only the antigens for *Pseudomonas* species (P-value = 0.0351) and Streptolysin O (P-value = 0.0074) showed a significant result.

**Conclusion:**

Although our results are limited by the lack of a control group and few antibody responses, they provide some evidence for infection with *Streptococcus A* as a potential contributing factor to ADL episodes. Our results add to the current evidence and illustrate the importance of determining the causal role of bacterial and fungal pathogens and immunological antifilarial response in ADL episodes.

## Introduction

Lymphatic filariasis (LF) is a neglected tropical disease affecting 68 million people throughout the world [1]. The disease is caused by 3 different species of parasitic nematode worms, *Wuchereria bancrofti*, *Brugia malayi*, and *Brugia timori* [2], which are spread by several genera of mosquitoes. These thread-like worms reside in the lymphatic vessels of humans, causing lymphatic damage.

While often characterized by chronic lymphedema and ultimately elephantiasis of the limbs, the first clinical sign of lower limb LF disease is typically an episode of acute adenolymphangitis (ADL). ADL is characterized by a plaque-like area of relatively diffuse cutaneous inflammation with or without ascending lymphangitis or satellite adenitis [3]. ADL episodes are accompanied by swelling, inflammation, high fever, general malaise and chills. The episodes are a recurrent clinical aspect of LF lasting 3–15 days each and may occur several times each year [4]. ADL episodes are often accompanied or followed by distal edema of the affected leg [3]. Clinical evidence has shown that ADL episodes can lead to the progression of chronic lymphedema [5–8]. Furthermore, ADL episodes have been shown to have a substantial economic impact through the loss of productive work days and the direct cost of treatment [9–11].

Despite extensive research, the etiology of ADL episodes is yet to be fully understood. Although there has been evidence for bacterial pathogens such as *Streptococcus A* and *Staphylococcus* as the causal agent in ADL episodes [12–16], other studies have attributed them to the host’s inflammatory/immune response to adult filarial worms in the lymphatic system or microfilariae in the blood [17,18]. Other studies suggest that filarial larvae likely contribute to observed ADL episodes [19] or have found associations between filarial intensity and ADL incidence [20]. Additionally, recent work has focused on the role of the *Wolbachia* endosymbiont in ADL episodes [21]. The role of fungal infections in predisposing to ADL episodes has also been explored [22,23]. Fungi can cause entry lesions, often between the toes and within deep skin folds[24], which serve as points of entry for bacteria.

Understanding the etiology and immunology of ADL episodes is important because they contribute to the progression of chronic LF—a major cause of disability worldwide. Our objective was to understand how antibody response to different antigens changes over the course of an ADL episode in order to inform models of ADL episode causality. To do this, we quantified antibody levels to pathogens that potentially contribute to ADL episodes during and after an episode among lymphedema patients in Léogâne, Haiti.

## Methods

### Study population

Between June 1995 and December 1997, lymphedema patients were enrolled in a study to test the feasibility and effectiveness of a basic lymphedema management program [25]. Patients were recruited at the outpatient clinic of Ste. Croix Hospital in Léogâne, Haiti, which is endemic for *Wuchereria bancrofti*. Patients were included if they had lymphedema of the leg, agreed to return to the clinic for follow-up evaluations, and did not have an obvious cause of edema such as a tumor. Data were collected on demographics, number and history of ADL episodes, and leg volume. Patients’ lymphedema was classified into stages using the 7-stage system of Dreyer and colleagues [26], and stages 4–7 were collapsed into a single fourth-stage category [25]. Of 302 patients who first received lymphedema care at the clinic during the enrollment period, 230 were enrolled. Of these, 175 returned to the clinic for at least 5 routine follow-up visits over a period of 6 months and were included in the original cohort. All patients continued through the end of the study until December 1998.

A subset of 41 patients of the original 175 patients was used for this analysis. In addition to the demographic, ADL, and leg volume information, serum samples were collected from these patients. During an ADL episode, patients were instructed to report to the clinic where a serum sample was taken. They were then instructed to return to the clinic after the ADL episode had ended for collection of a convalescent serum sample. Attempts were made by the investigators to obtain serum samples from all 175 patients enrolled in the original cohort, but not all patients were able to get to the hospital during an ADL episode as they may have been disabled and others did not consent to have their blood drawn. Thirty three of the 41 patients had serum samples taken during and following one ADL episode comprising 33 paired samples. Eight patients had serum samples during and following multiple ADL episodes: 3 of which had serum drawn during and after 3 episodes comprising 9 paired samples and 5 had serum drawn during and after 2 episodes comprising 10 paired samples. Overall there were a total of 52 paired samples and 104 observations. The current analyses were conducted post-hoc from 2013–2014.

### Ethics statement

The study was approved by the Ethics Committee of Ste. Croix Hospital and by the Institutional Review Board of the Centers for Disease Control and Prevention. To be eligible for participation, patients were required to provide written informed consent.

### Pathogen specific antigens

The samples collected from 1995–1998 were stored at -20°C at the CDC Laboratories in the Division of Parasitic Diseases and Malaria until testing was completed in 2000. We measured the antibody level for a variety of filarial, bacterial, and fungal pathogens hypothesized to be associated with ADL episodes. To test for filarial antibodies we used *Brugia pahangi* antigens that were extracted from adult worms as previously described [22]. These assays measure antibody responses to filarial antigens following *Brugia* and *Wuchereria* infections. Isotype-specific responses were measured with isotype-specific monoclonal antibodies. An IgG1 response to Brugia (BpG1) is considered to be a measure of filarial exposure [27], whereas BpG4 is a measure of filarial IgG4 antibody which is associated with active filarial infection [28] and is higher among microfilaremic patients (those with microfilariae circulating in the blood) as well as antigen-positive persons [29,30]. We also measured filarial IgG2 and IgG3 antibodies through the BpG2 and BpG3 antigen tests.

IgG antibodies to the fungal pathogens *Candida* and *Trichophyton* were measured using crude extracts from culture organisms. We measured IgG antibody to the bacterial pathogen *Streptococcus A* with several different antigens: the virulence factors Streptococcal Pyrogenic Exotoxin A (SPEA) and Streptococcal Pyrogenic Exotoxin B (SPEB), the hemolytic exotoxin Streptolysin O (SLO), and the carbohydrate antigen *Streptococcus* group A (StrepA). In addition to the streptococcal bacterial pathogens, we measured IgG responses to *Pseudomonas* using an exotoxin antigen and *Staphylococcus* using *Staphylococcus* enterotoxin B (SEB).

### Serologic assays

The Og4C3 ELISA assay [31]to detect circulating *Wuchereria bancrofti* antigen was used to determine which lymphedema patients were currently infected with the parasite. Assays to detect antibody response to adult filarial antigen were performed using biotinylated monoclonated antibodies (Zymed Laboratories, San Francisco, CA) specific for IgG1, IgG2, IgG3, and IgG4. Assays for antibody response to *Streptococcus* group A (Lee Laboratories, Grayson, GA), Streptolysin O, *Pseudomonas* exotoxin (List Biological Laboratories), *Staphylococcus* enterotoxin B (Sigma Chemical Co., St. Louis, MO), *Candida*, and *Trichophyton* were done using biotinylated monoclonal antibodies for total IgG. All antibodies were standardized against a high-titered serum sample included in each assay and arbitrarily assigned an antibody level of 10,000 units per ml. Antibody levels were then transformed into arbitrary units (AU). Plasma levels for all antigens were assayed in triplicate at a final dilution of 1:50. Acute and convalescent samples from a given patient were run on the same plate.

### Sufficient component cause models

In exploring potential filarial, bacterial, or fungal pathogens associated with ADL episodes, we conceptualized them using sufficient component cause models (SCCM) [32]. We assume that infection with these pathogens leads to an antibody response. These infections may or may not interact with a number of measureable factors such as age and gender and unknown factors such as genetics and the surrounding environment, ultimately leading to the ADL episode. In this framework, each risk factor for the outcome of interest is defined as a component cause or predisposing factor of the outcome of interest. Each component cause contributes to one or more sufficient causes, which contain the minimal set of conditions that result in the outcome [33–35]. An outcome may have a single or multiple sufficient causes.

**Fig 1:**
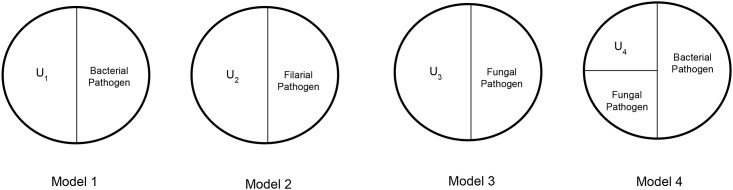
Sufficient component cause models (SCCMs) of ADL episodes among patients with lymphatic filariasis. Each SCCM represents a sufficient cause, which contains a minimal set of conditions that result in the outcome of interest. Each piece of the pie represents a risk factor for the outcome and is defined as a component cause. Each pie is defined as a sufficient cause.

Fig 1 displays potential SCCMs for ADL episodes. Model 1 states that at minimum, the presence of a bacterial infection such as *Streptococcus A* along with unknown factors U_1_ is sufficient to cause an episode of ADL. Model 2 states that at minimum, a reaction to filarial worm infection in combination with unknown factors U_2_ must be present for ADL episode to occur. Model 3 states that at minimum, the presence of a fungal infection such as *Trichyophtyon* in combination with unknown factors U_3_ is sufficient to cause an episode of ADL. Model 4 involves a biological interaction of infection with both a bacterial and fungal pathogen. The fungal pathogen causes a lesion on the skin of the affected limb, which then provides an entry point for bacteria and the subsequent response to infection in the form of an ADL episode. The unknown factors stated in these SCCMs may comprise a multitude of environmental, genetic, and immunologic factors. The examples of sufficient component causes displayed in Fig 1 represent only a few of the many potential sufficient cause models for ADL episodes.

### Antibody change assumptions

If infection by a particular pathogen immediately prior to an ADL episode contributes to the etiology of the episode, we expect the antibody levels to increase from the ADL to the convalescent time point [12,16,36]. We assumed that in the context of lymphedema patients with recurrent ADL episodes and bacterial/fungal infections, if the antibody level of a pathogen is found to increase two-fold from the ADL to the convalescent time point, it is considered a component cause of ADL.

### Statistical analysis

For each antigen, we calculated an absolute change and percent change from the ADL to convalescent time point:

$$Absolute\;Change\;=Antibody\;Level_{Convalescent}-Antibody\;Level_{ADL}$$

$$Percent\;Change\;=[(Antibody\;Level_{Convalescent}-Antibody\;Level_{ADL})/\left(Antibody\;Level_{ADL}\right)]\;X100$$

Since the distribution of antibody change in this population was not normal, we reported median values for the absolute antibody values, absolute change, and percent change along with the standard deviation and inter-quartile range (IQR). We conducted a Wilcoxon signed rank test to detect a difference between ADL and convalescent median antibody levels.

For each antigen we calculated the proportion of antibody response as the number of cases showing an antibody change at the two-fold level divided by the total number of ADL episodes in the sample. The denominator for the proportion is 52 ADL episodes.

$$Proportion\;of\;Two-Fold\;Antibody\;Response\;=\frac{\#\;of\;episodes\;showing\;two-fold\;change}{52\;ADL\;episodes}$$

## Results

The majority of the lymphedema patients were female (83%) with a median age of 34 years (Table 1). Most patients presented with stage 2 or 3 lymphedema. Only 1 of the 41 patients tested positive for circulating filarial antigen (CFA) at either the ADL or the convalescent time point. Prior to cohort enrollment, the yearly ADL rate among lymphedema patients was 2.7 per person year. During the entire lymphedema cohort enrollment, 1995–1998, the ADL rate reported was 1.42 per person year (109 episodes over 76.81 person years). Up until the ADL episode during which serum was collected, patients had experienced an average of 3 ADL episodes during their time enrolled in the lymphedema management program. The mean time between the ADL and convalescent serum sample was 17 days (range = 1–35). At the observed ADL episode during which acute serum was collected, about a third of patients were treated with an antibiotic, 70% had an enlarged lymph node, 62% presented with retrograde progression of inflammation, 75% presented with inflammation of the skin, and 81% had acute swelling at the ADL site.

**Table 1:** Demographic and clinical characteristics of lymphedema patients in Léogâne, Haiti. N = 41 patients with 52 ADL episodes.

	N (%)
**Patient Characteristics (N = 41)**	
Female gender	34 (82.9)
Age (median, SD)	34 (15.3)
No. legs	82
Lymphedema stage	
0	24 (29.3)
1	6 (7.3)
2	22 (26.8)
3	22 (26.8)
4–7	8 (9.8)
CFA(+): ADL Time Point	1 (1.8)
CFA(+): Convalescent Time Point	1 (1.8)
Mean yearly rate of ADL episodes reported in year prior to cohort enrollment ^a^	2.7
Mean yearly rate of ADL episodes reported during cohort period 1995–1998	1.42
Mean number of ADL episodes during study period up until serum sample ^b^	3.3
**ADL Episode Characteristics (N = 52)** ^c^	
Mean number of days between ADLA and convalescent sample (range)	17 (1–35)
Treated with antibiotic during episode	14 (26.9)
Lymph node enlargement	36 (69.2)
Retrograde progression of inflammation	32 (61.5)
Inflammation of skin (redness, pain, tenderness)	39 (75.0)
Acute swelling at ADL site	42 (80.8)

^a^ The study period of the lymphedema management effectiveness cohort = 1995–1998. This number represents the mean yearly rate of ADL episodes reported in the year prior to their enrollment. N = 38 (missing information on 3 patients)

^b^ The study period of the lymphedema management effectiveness cohort = 1995–1998. This number represents the number of ADL episodes from patient enrollment in the cohort up until the observed ADL episode during which serum was collected.

^c^ Serum samples were collected from 52 ADL episodes among the 41 lymphedema patients

CFA: circulating filarial antigen

### Two-fold antibody response

**Fig 2:**
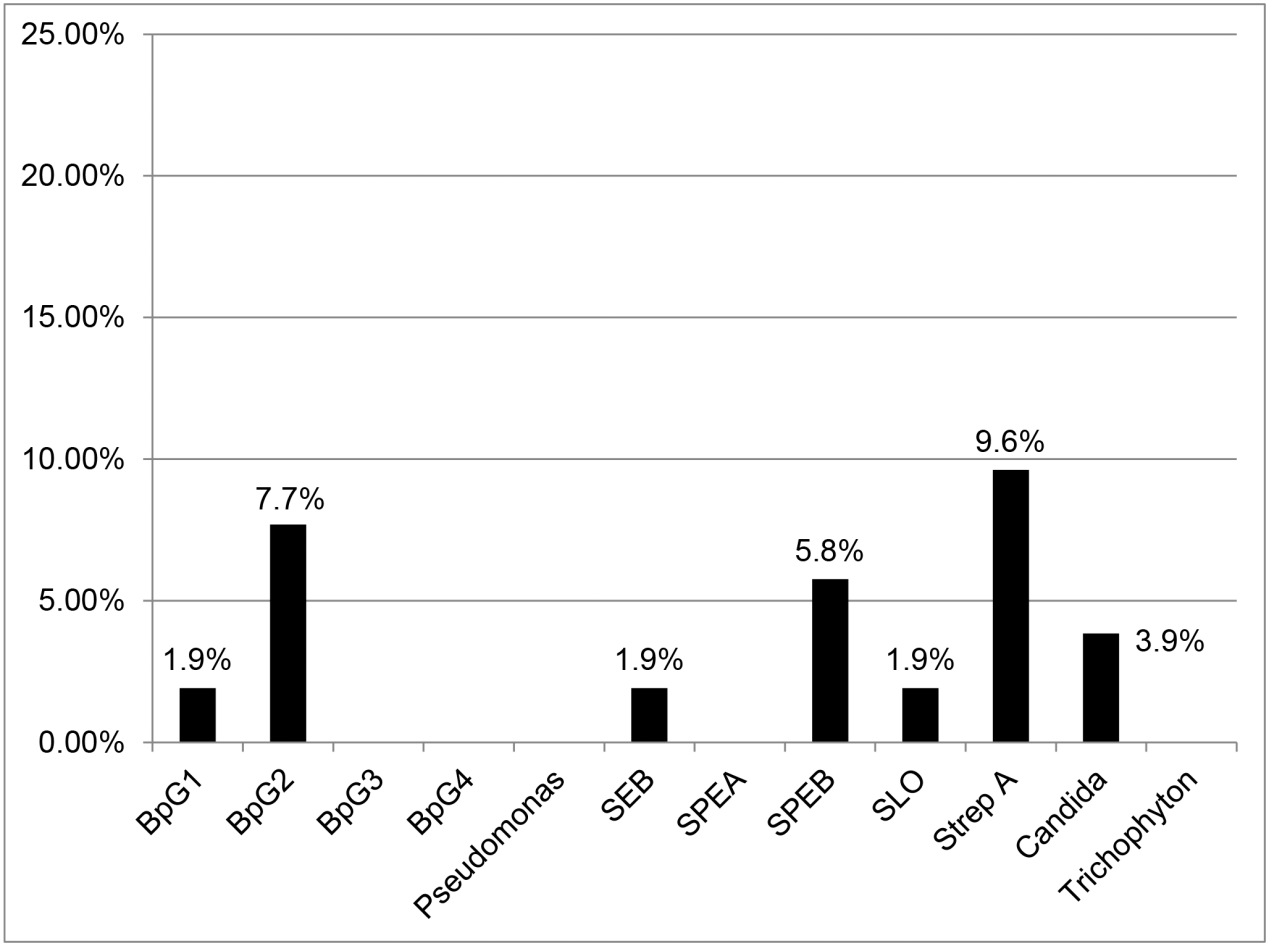
Proportion of ADL episodes with an antibody response for filarial, bacterial, and fungal antigens among a cohort of 52 paired serum samples from patients enrolled in a lymphedema program in Léogâne, Haiti. Filarial antigens: BpG1, BpG2, BpG3, and BpG4. Bacterial antigens: *Pseudomonas*, SEB, SPEA, SPEB, SLO, and Strep A. Fungal antigens: Candida and Trichophyton. All antibody change levels represent a 100 percent (two-fold) increase in antibody titer from the ADL to convalescent time point.

Fig 2 displays the proportion of ADL episodes with an antibody response for the filarial antigens (BpG1, BpG2, BpG3, and BpG4), bacterial antigens (SEB, SPEA, SPEB, SLO, and Strep A), and fungal antigens (*Candida* and *Trichophyton*). All responses represent a two-fold positive change of antibody level from the ADL to convalescent time point. Of the 52 paired serum samples, 5 (9.6%) showed an antibody response to the Strep A antigen, four (7.7%) showed a response to the BpG2 antigen, three (5.8%) showed a response to the SPEB antigen, and two (3.9%) showed a response to the *Candida* antigen. Only one (1.9%) paired sample demonstrated an antibody response to the BpG1, SEB, or SLO antigens. Our samples showed no response at the two-fold level to the BpG3, BpG4, *Pseudomonas*, and SPEA antigens.

Among the bacterial antigens, Strep A had the highest proportion of two-fold response. Among the filarial and fungal antigens, BpG2 and *Candida* had the highest proportions of response respectively.

### Serum antibody levels at ADL and convalescent time points

Table 2 displays the median antibody levels and standard deviations for each antigen during the observed ADL episode and convalescent time point with the corresponding p-value, median difference, and median percent change. All antibody levels are measured in arbitrary units (AU). When comparing the median antibody level during the ADL episode to the median antibody level at the convalescent time point for each antigen using a Wilcoxon signed rank test, only *Pseudomonas* (P-value = 0.0351) and SLO (P-value = 0.0074) showed a significant result. The median difference between paired ADL and convalescent time points ranged from -39.6 for BpG1 to 0.8 for SEB. The median % change between paired ADL and convalescent time points of greatest magnitude was seen for the SLO antigen (-9.6%). The median % change of smallest magnitude was for *Trichophyton* (-0.1%). SPEA had the greatest positive percent change (2.7%). Although the median % changes are minimal, the IQRs for each antigen are wide, indicating a great amount of variation.

**Table 2:** Serum antibody levels stratified by sample time point as determined by ELISA tests among lymphedema patients in Léogâne, Haiti. All values are in arbitrary units. N = 52 paired samples.

Indicator	ADL		Convalescent		P-value*	Median Absolute Difference (IQR)	Median % Change (IQR)
	Median	SD	Median	SD			
BpG1	1733.5	2934.2	1605.0	2629.3	0.7183	-39.6 (-209.5, 219.0)	-2.0 (-21.3, 13.3)
BpG2	707.9	2424.2	781.0	1767.4	0.5404	0.3 (-78.3, 131.0)	1.2 (-20.2, 25.9)
BpG3	57.1	140.6	50.4	220.0	0.7339	-1.3 (-7.1, 14.2)	-2.0 (-17.2, 20.2)
BpG4	88.8	1365.5	71.3	918.8	0.4325	-0.8 (-10.3, 10.1)	-2.8 (-10.4, 7.3)
Pseudomonas	38.1	76.1	36.9	66.3	0.0351	-1.6 (-5.5, 2.5)	-6.3 (-16.1, 7.0)
SEB	307.4	406.5	321.3	448.3	0.7117	-1.1 (-35.5, 33.1)	-0.5 (-12.1, 10.8)
SPEA	99.4	67.4	108.8	99.4	0.3656	0.8 (-15.3, 18.6)	2.7 (-15.2, 34.3)
SPEB	28.7	23.6	25.2	23.0	0.2384	-1.0 (-8.0, 5.5)	-3.2 (-30.8, 29.1)
SLO	132.6	123.8	135.8	126.8	0.0074	-12.1 (-38.5, 13.6)	-9.6 (-26.8, 16.6)
Strep A	130.4	154.7	150.1	146.6	0.8493	0.3 (-25.2, 32.2)	1.7 (-26.1, 32.5)
Candida	8.6	15.5	9.0	15.6	0.6290	0.0 (-0.7, 1.5)	-0.6 (-10.4, 18.6)
Trichophyton	67.3	45.9	64.7	46.3	0.9077	-0.2 (-7.0, 9.0)	-0.1 (-9.1, 12.5)

* Wilcoxon- signed rank test for median difference between ADL and convalescent antibody levels

IQR = inter-quartile range

## Discussion

To better understand factors associated with ADL episodes among lymphedema patients in Léogâne, Haiti, we quantified antibody levels to specific pathogens during and following an ADL episode. We conceptualized our hypotheses and will discuss our results in the context of sufficient component causal models (SCCM). We calculated the proportion of two-fold antibody responses for different pathogens hypothesized to be associated with ADL episodes. We assumed that those pathogens showing a two-fold change from an ADL to convalescent time point are considered a component cause of ADL episodes. Although a majority of the paired samples did not show a two-fold increase in anti-body levels for the antigens tested, our results showed the greatest proportion of two-fold changes in antibody levels for the Strep A antigen, followed by BpG2, SPEB, and *Candida*. Amongst the bacterial antigens, the Strep A antigen for *Streptococcus A* infection had the highest proportion of two-fold responses

Under our assumptions, these results support SCCM 1 (Fig 1) indicating that infection with the bacterial pathogen *Streptococcus A* in combination with one or many unknown factors (U_1_) could be sufficient to cause an episode of ADL. These findings are consistent with several other studies which found a high proportion of patients undergoing ADL to be infected with group A *Streptococcus*, leading to the conclusion that group A *Streptococcal* infection at least in part is associated with episodes of adenolymphangitis [12,13,16]. Of the two studies which also compared antibody levels at an ADL and convalescent time point, one found no significant rise in the antibody response to *Streptococcus A* [17], while the other found a significant rise in antistreptolysin O (ASO) titers from an ADL to convalescent time point [16]. The latter study also found that ADL patients had a higher mean ASO titer level than control patients, especially at the convalescent time point.

SCCM 2 states that at minimum, filarial worm infection in combination with a set of unknown factors U_2_ must be present for an ADL episode to occur. Although we saw responses for a change in filarial antibodies, the frequency of the filarial responses was less than the frequency of responses to *Streptococcus A* antigens, providing less evidence for the SCCM 2. Interestingly, the BpG2 filarial antigens had the highest proportion of change among the filarial antigens. Since IgG2 responses to filarial antigens are associated with carbohydrate epitopes, it is possible that these responses reflect cross reactivity stimulated by exposure to non-filarial antigens or even bacterial antigens. Responses to BpG4, indicating adult worm infection [28], would be most consistent with the SCCM 2, yet among CFA (-) lymphedema patients, the IgG4 responses are expected to be low [37,38] as seen in this cohort, thus adult worms are not likely to be the trigger for ADL. The second most frequent filarial response at the two-fold level was to the BpG1 antigen. Perhaps in our cohort of CFA (-) symptomatic LF patients, responses to BpG1 may also be indicative of SCCM 2, driven by exposure to L3 larvae. The BpG1 response is thought to be associated with exposure to filarial L3 larvae [27], but these may not necessarily result in patent infection. Combined with other unknown factors, the anti-filarial immune response to larval exposure may be sufficient to cause ADL episodes among this group of patients. Conversely, the anti-filarial response present in this CFA (-) cohort, though minimal, may in part be driven by cross-reactivity with fungal and bacterial pathogens and not due to the presence of filarial antibodies. Others have hypothesized that the antigen-negative filarial status of some lymphedema patients may be maintained, at least in part, by recurrent fungal and bacterial infections [22].

Our study also found that among the fungal antigens tested, the proportion of two-fold change was highest for the *Candida* antigen. This evidence would support SCCM 3 (Fig 1) where the presence of infection by a fungal pathogen such as *Candida* in combination with other unknown factors would potentially be sufficient for an ADL episode to occur. Previous studies have found evidence for the fungal pathogens as a contributing factor in the development of ADL episodes [22,23]. Fungal infections are often found between the toes and within deep skin folds, and can lead to inter-digital entry lesions [24]. These entry lesions can then serve as the point of entry for bacteria leading to the ADL episodes.

Although we only observed a statistically significant difference in the median antibody level between the ADL and convalescent samples for *Pseudomonas* and SLO (Table 2), the antibody levels were highly variable as indicated by the large standard deviations and wide inter-quartile ranges. The grouped analysis presented in Table 2 may not provide insights into the model of ADL pathogenesis; it instead illustrates the large amount of inter-subject variability present in this cohort. To explore this further, an analysis of variance with absolute antibody level for each antigen as the outcome and subject as the independent variable showed R^2^ values ranging from 0.83 to 0.98 (S1 Table) indicating that a large part of the variation of antibody values was due to subject. Conversely, an ANOVA with the absolute antibody level for each antigen as the outcome and sample time (ADL vs. convalescent) as the independent variable showed low R^2^ values, ranging from 0.0011 to 0.0398 (S1 Table), again indicating that much of the antibody response variability in our cohort was due to subject. The lack of variance between the acute and convalescent samples limits the conclusions that can be made from this study.

Among lymphedema patients who experience recurrent ADL episodes and have suffered the chronic effects of lymphedema for many years, antibody responses may take several months to wane following the initial peak after infection. Instead of oscillating antibody levels between ADL episodes as we hypothesized, they may increase and then plateau at those higher levels, indicating a constant heightened immune response. In a comparison of ADL patients with healthy controls in the Dominican Republic, Vincent concluded that the mean anti-streptolysin O and anti-DNAse B titer levels of the affected persons may not fully return to normal between episodes [16].

In this context, where patients may have recurrent episodes, the threshold of antibody change may be lower than standard levels. Therefore, we assumed that a two-fold change in antibody level from an ADL to convalescent time point may be indicative of infection with the pathogen of interest. We did however, explore antibody level changes at the three-fold (200%) and four-fold (300%) levels, and found that few changed by this magnitude. We also explored the proportion of response at lower levels, but are not confident those levels of change are reproducible. The lack of antibody titer change from an ADL to convalescent time point at the higher levels may be due to several factors. The study population in Léogâne, Haiti is regularly exposed to the pathogens of interest and may already have elevated antibody titers. Therefore, a smaller rise in antibody levels from the ADL episode to the convalescent time point may be indicative of infection in this population.

There are several limitations in the design of this study. First, the sample size of 41 lymphedema patients is relatively small, limiting the external validity of our findings. Second, the 41 ADL patients who gave blood during and following an ADL episode may not have been representative of the original cohort of 175 lymphedema patients. Although there were no differences in the gender and age distribution, the distribution of lymphedema stage, duration of lymphedema and rate of ADL episodes reported in the year prior to study enrollment, the 41 ADL patients had a statistically significant higher rate of ADL episodes during the entire study period (1995–1998): 1.42 per person year among patients who provided serum vs. 0.75 per person year among original cohort patients (p-value <0.0001) (S2 Table). The more frequent occurrence of ADL episodes among this subset of the original cohort may have made these patients more willing to give blood, so that our findings may not be representative of others that did not volunteer to give blood.

Third, all antibody values are based on arbitrary units and not on a calibrated reference scale. Therefore, we can only explore absolute and relative differences in antibody levels between the ADL and convalescent samples and assume certain levels of change indicate infection. Furthermore, it is possible that blood was not drawn from individuals during the most florid period of their ADL episode. Patients were required to provide their own transportation to and from the clinic. Those who did willingly make the trip to the clinic to give blood may have done so after the most severe symptoms of their ADL episode had passed. If this is the case, the antibody levels measured during the ADL episode are probably an overestimate of the levels during the most florid time (assuming antibody levels increase over the period of interest). Thus, the differences in antibody levels from ADL to convalescent time point were probably an underestimate. Also, because we chose which pathogens to test *a priori*, we may have missed some component causes of ADL, which may contribute greatly to the ADL episodes in this study population. Because most of the variance in the antibody levels was due to subject and not due to time, our conclusions about which pathogens contributed the most to ADL episodes in this population based on arbitrary responses of change may not be as strong as findings from other studies in which a control population (no ADL episode) was present.

Lastly, we only collected information from 1–3 ADL episodes among each individual in this sample population. The lymphedema patients in the cohort have been exposed to LF for many years and may have experienced many ADL episodes throughout their lives. We assume that the pathogen(s) with the greatest contribution to ADL episodes at this time point will be typical of all ADL episodes in this population.

Through a descriptive analysis of the proportion of antibody response at a two-fold level of change, this study provides further, yet limited evidence for infection with *Streptococcus A* as a potential contributing factor of ADL episodes. However, such changes were only observed for a small proportion of ADL episodes and the contribution of *Streptococcus A* to ADL episodes in this cohort may be minimal compared to other unmeasured factors. The overall lack of response of the paired samples may suggest that none of the pathogens tested in this study are a major contributing factor to ADL episodes. Nonetheless, these findings are important within the context of previous research suggesting a relationship between ADL episodes and *Streptococcous* A and within the more recent focus on LF morbidity control in endemic areas. This work adds to the current evidence and illustrates the importance of determining the causal role of bacterial and fungal pathogens and immunological antifilarial response in ADL episodes.

## Supporting Information

S1 Table Variation of serum antibody levels due to subject, time, and time conditional on subject among lymphedema patients in Léogâne, Haiti. N = 104 samples.
(PDF)

S2 Table Demographic and clinical characteristics of lymphedema patients in Léogâne, Haiti, comparing the entire cohort to the ADL cohort. (PDF)
