# Supplementary material for: Changes in Antibody Levels during and following an Episode of Acute Adenolymphangitis (ADL) among Lymphedema Patients in Léogâne, Haiti
Source: PLoS One. 2015 Oct 22;10(10):e0141047. doi: 10.1371/journal.pone.0141047 (PMC4619626; doi:10.1371/journal.pone.0141047)
Supplement: S1 Table — N = 104 samples. (PDF) [file pone.0141047.s001.pdf]

S1 Table. Variation of serum antibody levels due to subject, time, and time conditional on subject among lymphedema patients in Léogâne, Haiti. N=104 samples.

Indicator	R^2 Subject	R^2 Time	R^2 Time Conditional on Subject
BpG1	0.93	0.00008	0.0011
BpG2	0.87	0.00224	0.0166
BpG3	0.90	0.00164	0.0215
BpG4	0.96	0.00111	0.0270
Candida	0.90	0.00141	0.0137
Pseudomonas	0.98	0.00163	0.0628
SEB	0.99	0.00003	0.0026
SPEA	0.92	0.01080	0.0465
SPEB	0.83	0.00252	0.0148
SLO	0.95	0.00204	0.0398
Strep A	0.89	0.00014	0.0013
Trichophyton	0.98	0.00008	0.0037
