# Supplementary material for: Changes in Antibody Levels during and following an Episode of Acute Adenolymphangitis (ADL) among Lymphedema Patients in Léogâne, Haiti
Source: PLoS One. 2015 Oct 22;10(10):e0141047. doi: 10.1371/journal.pone.0141047 (PMC4619626; doi:10.1371/journal.pone.0141047)
Supplement: S2 Table — (PDF) [file pone.0141047.s002.pdf]

S2 Table. Demographic and clinical characteristics of lymphedema patients in Léogâne, Haiti, comparing the entire cohort to the ADL cohort.

**Patient Characteristics (N=175)**	**Entire Cohort (n=175)**	**ADL Cohort[a] (n=41)**	**P-value**
Female gender	145 (82.9)	34 (82.9)	0.85
Age (median, SD)	38 (15.96)	34 (15.3)	0.09
% Literate	87 (49.7)	18 (43.9)	0.79
Mean (range) duration of lymphedema upon entry into study (years)	11.3 (<1-50)	10.8 (<1-37)	0.77
No. legs	350	82	
Lymphedema stage			
0 (No Edema)	85 (24.3)	24 (29.3)	0.1169
1	38 (10.8)	6 (7.3)	
2	129 (36.9)	22 (26.8)	
3	85 (24.3)	22 (26.8)	
4	13 (3.7)	8 (9.8)	
Yearly rate of ADL episodes reported in year prior to cohort enrollment[b]	2.06	2.7	0.1368
Yearly rate of ADL episodes reported during cohort period 1995-1998[c]	0.75	1.42	<0.0001

[a]Serum samples were collected from 52 ADL episodes among the 41 lymphedema patients

[b] The study period of the lymphedema management effectiveness cohort = 1995 – 1998. This number represents the mean yearly rate of ADL episodes reported during their enrollment. N=38 (missing information on 3 patients)

[c] The study period of the lymphedema management effectiveness cohort = 1995 - 1998. This number represents the number of ADL episodes from patient enrollment in the cohort up until the observed ADL episode during which serum was collected.

CFA: circulating filarial antigen
